# A Non-SOGI Single-Phase Frequency-Locked Loop Based on Synchronous Frequency Tracking Filter with Enhanced Immunity to Sensor-Induced DC Offsets

**DOI:** 10.3390/s26206499

**Published:** 2026-10-14

**Authors:** Fehmi Sevilmiş

**Affiliations:** Department of Electrical and Electronics Engineering, Selçuk University, 42130 Konya, Türkiye; fehmisevilmis@selcuk.edu.tr

**Keywords:** complex bandpass filter (CBF), frequency-locked loop (FLL), second-order generalized integrator (SOGI), single-phase grid-connected converters, SOGI-FLL

## Abstract

In single-phase grid-connected converters, a second-order generalized-integrator-based frequency-locked loop (SOGI-FLL) is widely used to meet synchronization requirements due to its structural simplicity. However, since the quadrature-phase form of the input signal in this method exhibits a low-pass filter characteristic, its immunity to harmonic distortion and sensor-induced DC-offset errors is limited. To address these problems and improve synchronization accuracy under weak grids, this study proposes a non-SOGI frequency-locked loop (FLL) method based on a synchronous frequency tracking filter (SFTF), hereinafter referred to as SFTF-FLL. The most significant advantage of the suggested method compared to the standard SOGI-FLL is that its quadrature component exhibits a bandpass filter characteristic. This characteristic enables the SFTF-FLL to attenuate harmonic components and, more importantly, significantly suppress sensor-induced DC-offset errors in the input signal, thereby improving the accuracy of frequency estimation. To facilitate the parameter design of the suggested method, its small-signal model is derived. Comparative experimental results are provided with SOGI-FLL and complex bandpass filter-based FLL (CBF-FLL) to demonstrate the effectiveness and performance enhancement of the suggested SFTF-FLL. The numerical results demonstrate improved amplitude and phase estimation under harmonically distorted, sensor-induced DC-offset, and measurement noise conditions.

## 1. Introduction

The integration of power electronics converter-based renewable energy sources, which are rapidly replacing traditional energy generation systems, has fundamentally transformed the dynamics of modern power systems [1,2]. This transformation has led to a reduction in grid inertia and, consequently, more abrupt fluctuations in grid frequency and voltage sags [3,4]. Under such variable grid conditions, a fast and accurate synchronization algorithm is critical for grid-connected inverters to operate stably and efficiently [5,6,7,8]. However, adverse conditions commonly encountered in weak grid structures—such as voltage transients, harmonic distortions, and sensor-induced DC-offset components—severely challenge the performance of existing synchronization algorithms [9,10,11].

Recent studies have addressed disturbance mitigation in power-electronic converter systems at different levels. These include PWM-frequency harmonic suppression using a zero-vector switching SVPWM strategy [12], leakage-current attenuation using a component-reuse hybrid filter [13], and waveform shaping through a SiC device-based external module in modular multilevel converters [14]. Although these approaches focus on converter modulation, filtering, and topology rather than synchronization, they highlight the broader importance of disturbance attenuation in converter-based applications. Nevertheless, converter-based solutions do not eliminate measurement-based disturbances encountered by synchronization units, thereby motivating the development of robust synchronization methods capable of accurate fundamental-component extraction under adverse input conditions.

In the literature, phase-locked loops (PLLs) and frequency-locked loops (FLLs) are widely used for synchronization [15,16,17,18]. While PLLs typically focus on eliminating the phase difference between the input and output in a synchronous reference frame, FLLs operate in a stationary reference frame and directly track the frequency. In recent years, FLL methods have come to the forefront in academic research. This is because they do not require PI controllers, are very resistant to sudden phase jumps, and have simple architectures that do not require coordinate transformations, such as the Park transformation [19].

In single-phase systems, second-order generalized-integrator (SOGI)-based FLL (referred to as SOGI-FLL) is considered a standard single-phase FLL method due to its simple structure [20]. The SOGI structure extracts the fundamental component from the input signal while simultaneously generating the quadrature form of the input signal required for frequency extraction; however, the standard SOGI-FLL has limited filtering capability.

In particular, the quadrature form of the input signal in the SOGI structure exhibits a low-pass filter (LPF) characteristic. This leads to incorrect determination of the input signal’s amplitude, frequency, and phase when the input signal contains harmonic and sensor-induced DC-offset components [9,18,21,22]. To this end, in the literature, many advanced FLL architectures have been introduced to enhance the limited filtering ability and dynamic performance of SOGI-FLL.

The multiple-SOGI-based method (i.e., MSOGI-FLL), which incorporates structures connected in parallel to selectively suppress specific harmonic components, has been developed [23]. Although MSOGI-FLL is effective at filtering low-order harmonics, it requires the use of a significant number of parallel units, particularly in weak grids where the harmonic components are unknown. This makes the computational load even heavier and complicates the design of the parameters. Similarly, prefiltered and in-loop filtered SOGI-FLL architectures have been proposed to improve the system’s harmonic filtering capability and DC-offset immunity [21,24]. These architectures offer better harmonic suppression characteristics compared to the standard SOGI-FLL; however, they reduce the system’s phase margin, thereby narrowing the stability limits, causing larger overshoots during transient conditions, and slowing down the dynamic response [25]. Linear Kalman filter (LKF)-based FLLs offer high robustness in the presence of noisy signals, but they exhibit poor harmonic filtering performance compared to standard SOGI-FLL and require high computational load due to the continuous updating of Kalman gains. Transfer-delay-based adaptive FLL (TD-AFLL) structures are capable of providing a fast dynamic response [26,27]. Nonetheless, these methods have poor filtering capabilities and are extremely sensitive to sensor-induced DC-offset components, which limits their use in practical applications. In [28], Comb-FLL has been presented to eliminate harmonics and the sensor-induced DC-offset component. This FLL effectively eliminates the distortions in the input signal, but it has disadvantages due to its requirement for large memory capacity at high sampling frequencies and complexities such as rounding errors in digital implementations. Furthermore, while the extremum-seeking-based FLL (ES-FLL) proposed in [29] and the fourth-order generalized integrator-FLL (FOGI-FLL) proposed in [30,31] improve the disturbance rejection capability, they require complex tuning procedures. Another important alternative approach is the complex bandpass filter-based FLL (CBF-FLL) method [32]. The CBF-FLL treats the single-phase input signal as an unbalanced system and attempts to filter out the negative-sequence component. Although it can provide better filtering than standard SOGI-FLL, the filter bandwidth must be narrowed to completely eliminate harmonic oscillations; this causes the system to respond more slowly.

Although the aforementioned methods improve particular aspects of single-phase synchronization, they employ different mechanisms for fundamental-component extraction, quadrature-signal generation, and disturbance rejection. To clarify the structural position of the proposed method with respect to the closest existing FLL architectures, their main characteristics are provided in Table 1. As summarized in Table 1, standard and enhanced SOGI-FLL methods retain the generalized-integrator resonant core, whereas their filtering capability is improved using additional filters or parallel harmonic-selective units. Other non-SOGI-based FLLs achieve disturbance rejection through complex filtering, time delays, stored data windows, or multiple resonant units. In contrast, the proposed structure first extracts the fundamental component using a synchronous frequency tracking filter (SFTF) and then generates the quadrature component from this filtered signal using a frequency-adaptive all-pass filter (APF). Consequently, both orthogonal signals exhibit the characteristics of a bandpass filter.

In this paper, to deal with the shortcomings of existing FLL approaches, a non-SOGI single-phase synchronous frequency tracking filter (SFTF)-based FLL (shortly named the SFTF-FLL) is proposed. The SFTF-FLL consists of an SFTF unit for extracting the fundamental component and an all-pass filter (APF) unit for generating the quadrature component of the input signal. The main contributions of the proposed SFTF-FLL compared to existing FLL methods can be summarized as follows:

- The SFTF is applied for the first time as the fundamental-component extraction stage of a single-phase FLL-based grid-synchronization method.
- An integrated SFTF–APF–FLL architecture is developed, in which the quadrature component of the input signal is generated from the filtered SFTF output using a frequency-adaptive APF.
- Both in-phase and quadrature components exhibit bandpass characteristics, enhancing DC-offset and harmonic suppression compared with existing methods such as SOGI-FLL.

The performance of the suggested SFTF-FLL is experimentally evaluated by comparing it with the SOGI-FLL and CBF-FLL methods. The findings indicate that the suggested SFTF-FLL is a more effective solution for single-phase systems compared to the existing FLL methods.

## 2. Standard SOGI-FLL

**Figure 1:**
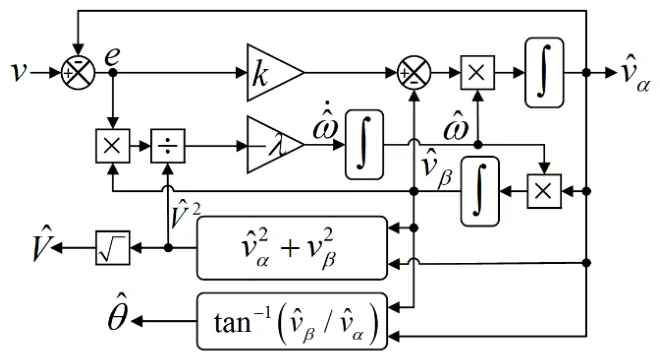
Structure of the standard SOGI-FLL.

Figure 1 illustrates the block diagram of the standard single-phase SOGI-FLL [18], where *v* represents the input signal, and ${\hat{v}}_{\alpha }$ and ${\hat{v}}_{\beta }$ represent the filtered in-phase and quadrature-phase forms of *v*, respectively. $\hat{\omega }$, $\hat{V}$, and $\hat{\theta }$ express the estimated frequency, amplitude, and phase angle of the input signal, respectively. *k* and *λ* stand for the SOGI and FLL gains, respectively. By disregarding the FLL dynamics, the transfer functions of SOGI in the *s*-domain are as follows [33].

(1) $$G_{\alpha }(s)=\frac{{\hat{v}}_{\alpha }(s)}{v(s)}=\frac{k\hat{\omega }s}{s^{2}+k\hat{\omega }s+{\hat{\omega }}^{2}}$$

(2) $$G_{\beta }(s)=\frac{{\hat{v}}_{\beta }(s)}{v(s)}=\frac{\hat{\omega }}{s}G_{\alpha }(s)=\frac{k{\hat{\omega }}^{2}}{s^{2}+k\hat{\omega }s+{\hat{\omega }}^{2}}$$

**Figure 2:**
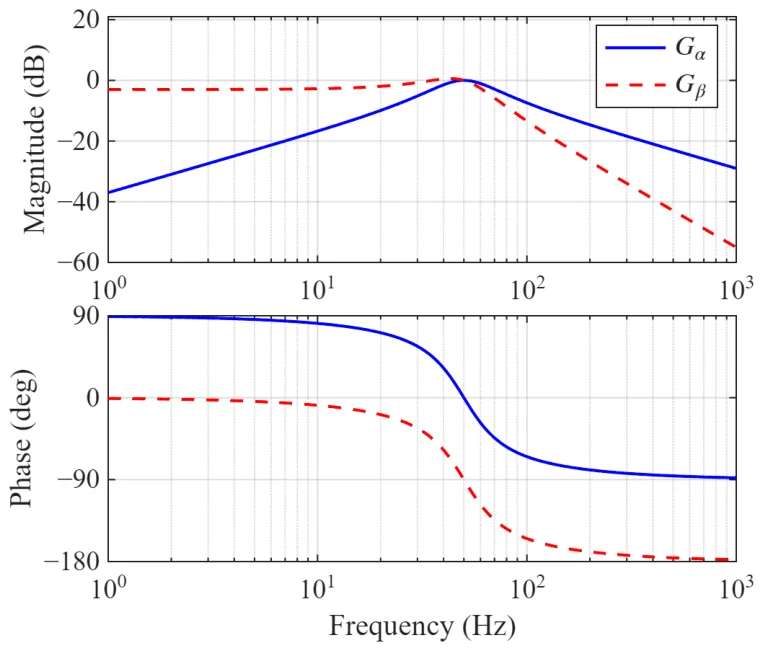
Bode diagrams of *G_α_*(*s*) and *G_β_*(*s*) for SOGI-FLL.

Figure 2 shows the Bode diagrams of the transfer functions *G_α_*(*s*) and *G_β_*(*s*), in which $k=1/\sqrt{2}$ and $\hat{\omega }=2\pi 50$ rad/s. As shown, *G_α_*(*s*) acts as a bandpass filter, which provides a unity gain and a zero phase at 50 Hz. It indicates that ${\hat{v}}_{\alpha }$ is a filtered in-phase form of the input signal *v*. However, *G_β_*(*s*) behaves as a low-pass filter with a unity gain and −90° phase at 50 Hz. It means that ${\hat{v}}_{\beta }$ signifies a filtered quadrature-phase form of the input signal *v*.

The parameter design process for nonlinear systems can be very challenging or nearly impossible. To perform parameter design, a small-signal model that closely tracks the responses of the nonlinear system is often used. To tune the FLL gain *λ* and evaluate the stability, it is quite useful to obtain the small-signal model of SOGI-FLL. Although the modeling procedure of the standard SOGI-FLL has already been discussed in many papers such as [18,21], its small-signal model is briefly obtained in this study. This small-signal model simplifies the modeling procedure of the proposed method.

To facilitate the modeling procedure, harmonics in the input signal can be ignored, and the input signal is expressed as:

(3) $$v(t)=V\cos(\underset{\theta }{\underset{︸}{\mathrm{\omega t}+\varphi }})$$

where *V*, *ω*, *φ*, and *θ* symbolize the amplitude, angular frequency, initial phase angle, and phase angle, respectively. Based on the Bode plots in Figure 2, the output signals of SOGI (i.e., ${\hat{v}}_{\alpha }$ and ${\hat{v}}_{\beta }$) can be taken into consideration as:

(4) $${\hat{v}}_{\alpha }(t)=\hat{V}\cos(\hat{\theta })$$

(5) $${\hat{v}}_{\beta }(t)=\hat{V}\sin(\hat{\theta })$$

In accordance with the modeling procedure of SOGI-FLL, it is assumed that $\hat{V}\approx V$, $\hat{\omega }\approx \omega$, and $\hat{\theta }\approx \theta$ in a quasi-locked state. Considering the SOGI-FLL structure in Figure 1, the differential equations can be defined as:

(6) $$\overset{•}{\hat{\omega }}=-\frac{\lambda }{{\hat{V}}^{2}}{\hat{v}}_{\beta }(\overset{e}{\overset{︷}{v-{\hat{v}}_{\alpha }}})$$

(7) $$\overset{•}{{\hat{v}}_{\alpha }}=\hat{\omega }{\hat{v}}_{\beta }(\mathrm{kv}-k{\hat{v}}_{\alpha }-{\hat{v}}_{\beta })$$

(8) $$\overset{•}{{\hat{v}}_{\beta }}=\hat{\omega }{\hat{v}}_{\alpha }$$

Substituting (3)–(5) into (6) and applying some mathematical manipulations yields:

(9) $$\begin{array}{l} \overset{•}{\hat{\omega }}=-\frac{\lambda }{{\hat{V}}^{2}}\hat{V}\sin(\hat{\theta })\left[V\cos(\theta )-\hat{V}\cos(\hat{\theta })\right]\\ =\frac{\lambda }{2\hat{V}}\left[\underset{\approx 0}{\underset{︸}{\hat{V}\sin(2\hat{\theta })-V\sin(\theta +\hat{\theta })}}+\underset{\approx (\theta -\hat{\theta })}{\underset{︸}{V\sin(\theta -\hat{\theta })}}\right]\\ \approx \frac{\lambda }{2}(\theta -\hat{\theta }) \end{array}$$

From Figure 1, the estimated phase angle is:

(10) $$\hat{\theta }=\tan^{-1}\left(\frac{{\hat{v}}_{\beta }}{{\hat{v}}_{\alpha }}\right)$$

The differentiation of (10) with respect to time results in:

(11) $$\overset{•}{\hat{\theta }}=\frac{\overset{•}{{\hat{v}}_{\beta }}{\hat{v}}_{\alpha }-\overset{•}{{\hat{v}}_{\alpha }}{\hat{v}}_{\beta }}{{\hat{v}}_{\alpha }^{2}+{\hat{v}}_{\beta }^{2}}=\frac{\overset{•}{{\hat{v}}_{\beta }}{\hat{v}}_{\alpha }-\overset{•}{{\hat{v}}_{\alpha }}{\hat{v}}_{\beta }}{{\hat{V}}^{2}}$$

Substituting (7) and (8) into (11) gives:

(12) $$\overset{•}{\hat{\theta }}=\frac{\hat{\omega }(\overset{{\hat{V}}^{2}}{\overset{︷}{{\hat{v}}_{\alpha }^{2}+{\hat{v}}_{\beta }^{2}}})-k\hat{\omega }\overset{-\dot{\hat{\omega }}{\hat{V}}^{2}/\lambda }{\overset{︷}{(v-{\hat{v}}_{\alpha }){\hat{v}}_{\beta }}}}{{\hat{V}}^{2}}=\hat{\omega }+\frac{k\hat{\omega }}{\lambda }\overset{•}{\hat{\omega }}$$

As observed from (12), the coefficient of $\overset{•}{\hat{\omega }}$ provides a time-variant term, which means that it is not possible to realize the Laplace transform of (12). To deal with this challenge, using a linear time-invariant (LTI) model, the estimated frequency in this coefficient is considered to be the nominal frequency *ω_n_* as follows [21].

(13) $$\overset{•}{\hat{\theta }}\approx \hat{\omega }+\frac{k\omega_{n}}{\lambda }\overset{•}{\hat{\omega }}$$

**Figure 3:**
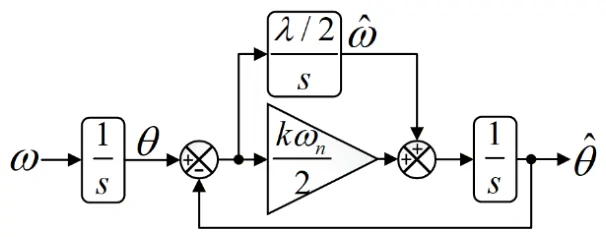
Small-signal model of SOGI-FLL.

The small-signal model of the SOGI-FLL is derived from (9) and (13), as illustrated in Figure 3.

In order to confirm the accuracy of the small-signal model, the following two simulation tests are performed.

Test-1: A 20° phase jump occurs.

Test-2: A +2 Hz frequency jump occurs.

**Figure 4:**
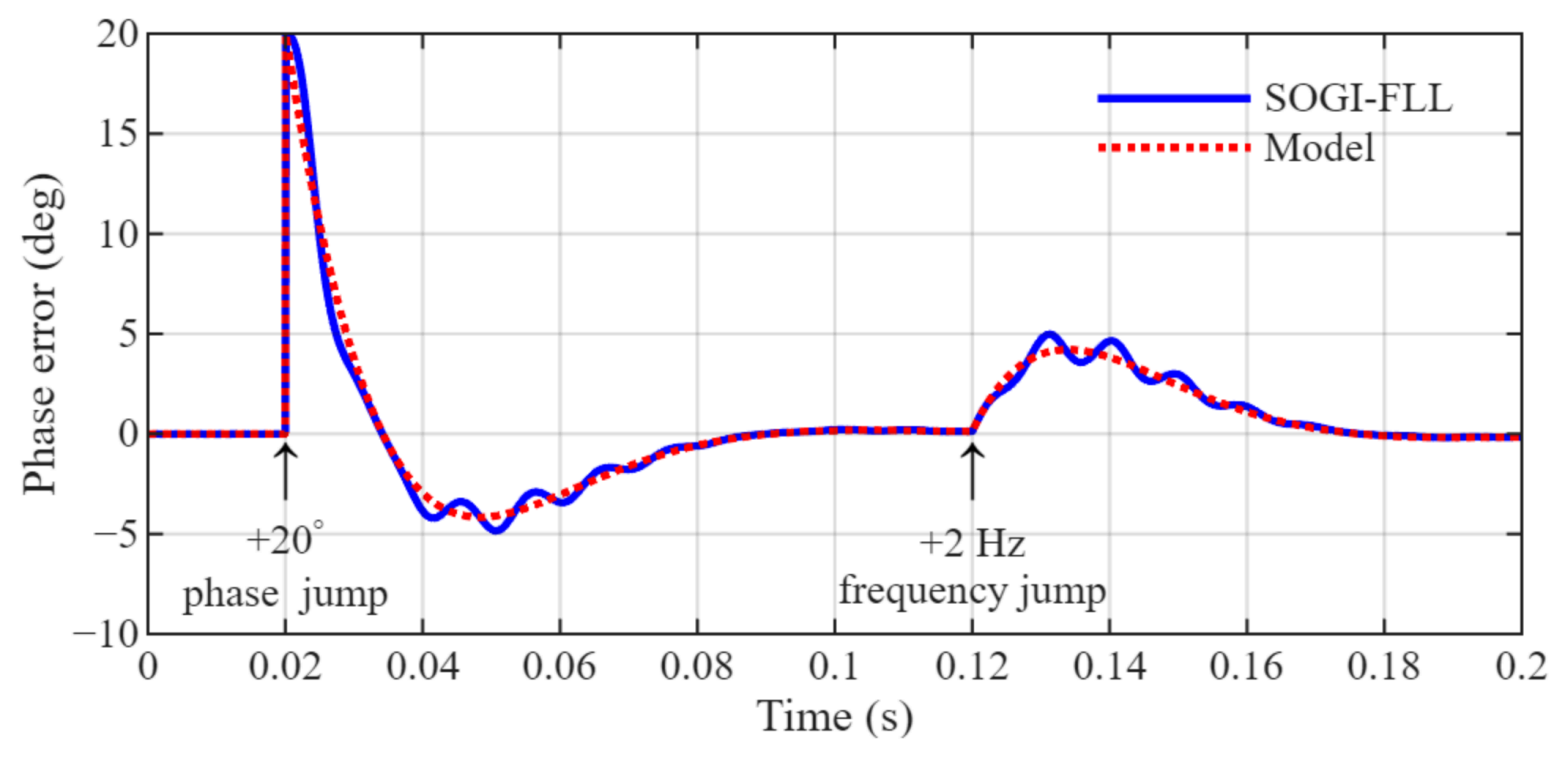
Accuracy evaluation of the small-signal model of SOGI-FLL.

In these tests, the estimated phase angle error by the SOGI-FLL as in Figure 1 is compared with that of its small-signal model as in Figure 3. Figure 4 shows the results of Test-1 and Test-2. As observed, the small-signal model accurately predicts the average dynamic behavior of the SOGI-FLL.

Based on Figure 3, the closed-loop transfer functions of the SOGI-FLL are expressed as:

(14) $$\frac{\hat{\omega }(s)}{\omega (s)}=\frac{\lambda /2}{s^{2}+(k\omega_{n}/2)s+\lambda /2}$$

(15) $$\frac{\hat{\theta }(s)}{\theta (s)}=\frac{(k\omega_{n}/2)s+\lambda /2}{s^{2}+(k\omega_{n}/2)s+\lambda /2}$$

To set the control parameter of SOGI-FLL (i.e., *λ*), the characteristic equation of (14) and (15), which is a standard second-order system, is used as:

(16) $$s^{2}+\underset{2\xi \omega_{n}^{\prime }}{\underset{︸}{(k\omega_{n}/2)}}s+\underset{{\left(\omega_{n}^{\prime }\right)}^{2}}{\underset{︸}{\lambda /2}}=0$$

where *ξ* is the damping factor and $\omega_{n}^{\prime }$ is the natural frequency.

Using (16), the following equations can be obtained as:

(17) $$\omega_{n}^{\prime }=\frac{k\omega_{n}}{4\xi }$$

(18) $$\lambda =2{\left(\omega_{n}^{\prime }\right)}^{2}$$

Substituting (17) into (18) results in:

(19) $$\lambda =\frac{k^{2}\omega_{n}^{2}}{8\xi^{2}}$$

From (19), *λ* is determined by selecting *ξ* and *k*. Please note that the nominal frequency of the input signal *ω_n_* is defined before as 2*π*50 rad/s. The damping factor *ξ* is recommended as $1/\sqrt{2}$ in many control papers to achieve an optimum trade-off between the settling time and overshoot [21,34]. The SOGI gain *k* affects both the filtering capability and dynamic response of SOGI-FLL. To accelerate its dynamic response, *k* is increased, but this leads to a low filtering capability. Therefore, it is properly tuned to ensure a trade-off between the filtering capability and dynamic response. As recommended in [21], *k* is chosen as $1/\sqrt{2}$ in this paper. As a result, the selected values *ξ* and *k* are substituted into (19), and *λ* = 12,337 is obtained.

## 3. Proposed SFTF-FLL

**Figure 5:**
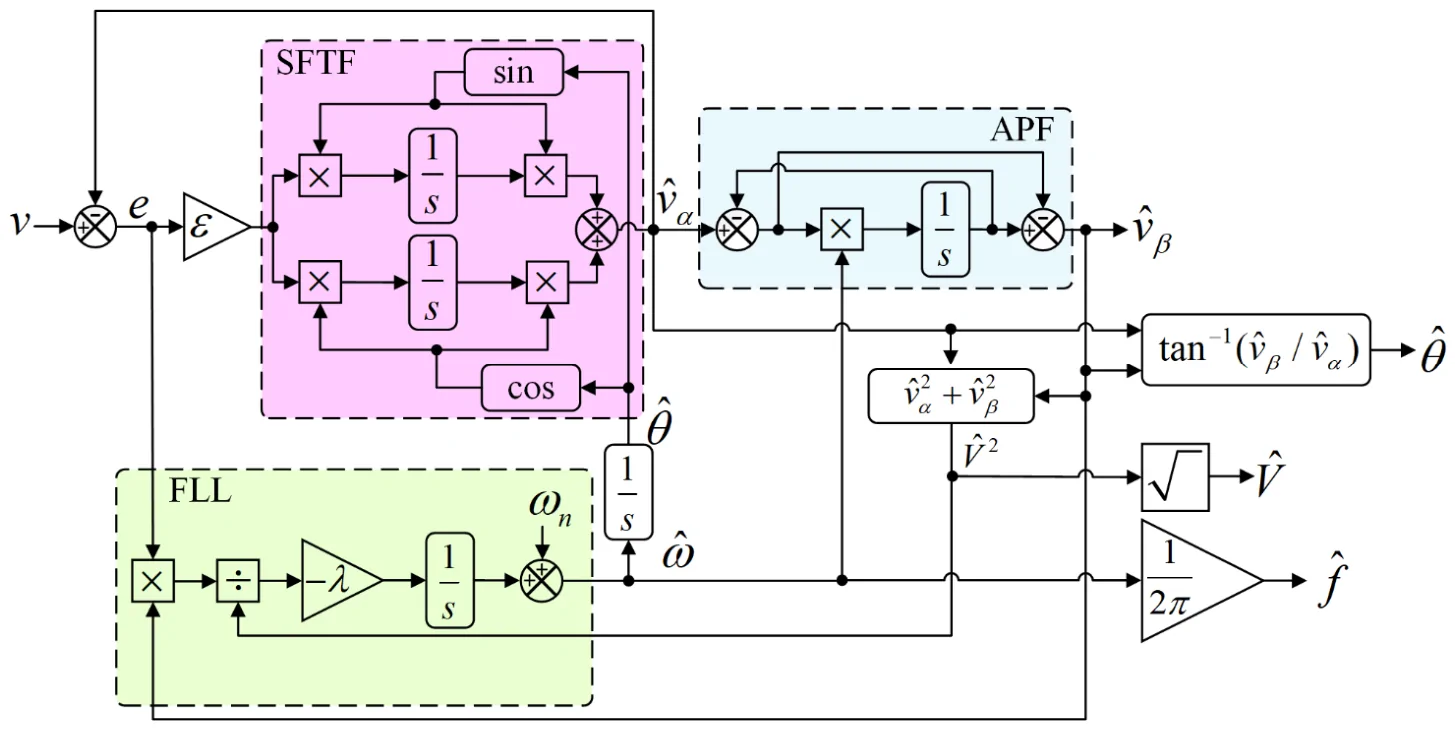
Block diagram of the proposed SFTF-FLL.

Figure 5 illustrates the proposed non-SOGI-based FLL method, called SFTF-FLL. It consists of the SFTF, APF, and FLL structures, which are explained in this section.

### 3.1. SFTF Structure

**Figure 6:**
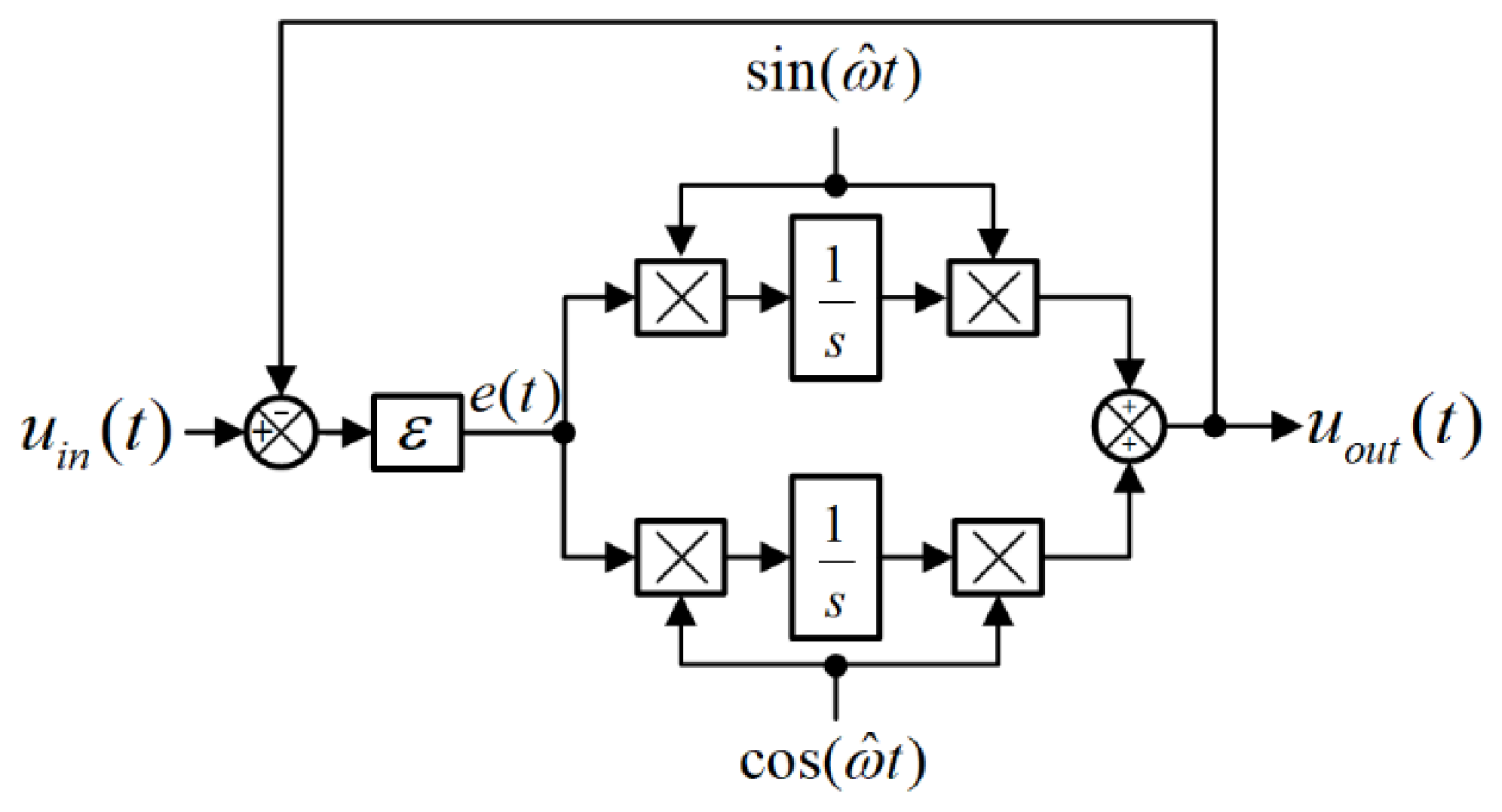
Block diagram of the SFTF.

Figure 6 depicts the block diagram of the SFTF [35], where *u_in_*(*t*) and *u_out_*(*t*) are its input and output signals, respectively, and *ε* is the proportional gain. From Figure 6, *u_out_*(*t*) can be defined as:

(20) $$u_{\mathrm{out}}(t)=\sin\left(\hat{\omega }t\right)\left\{\int \sin\left(\hat{\omega }t\right)e\left(t\right)\mathrm{dt}\right\}+\cos\left(\hat{\omega }t\right)\left\{\int \cos\left(\hat{\omega }t\right)e\left(t\right)\mathrm{dt}\right\}$$

where $\hat{\omega }$ is the estimated angular frequency and is defined as $\hat{\omega }=d\hat{\theta }/\mathrm{dt}$. Taking the second derivative of (20) yields:

(21) $${\ddot{u}}_{\mathrm{out}}(t)+{\hat{\omega }}^{2}u_{\mathrm{out}}(t)=\dot{e}(t)$$

Based on (21) and Figure 6, the closed-loop transfer function of SFTF can be stated in the *s*-domain as:

(22) $$G_{\mathrm{SFTF}}(s)=\frac{u_{\mathrm{out}}(s)}{u_{\mathrm{in}}(s)}=\frac{\mathrm{\varepsilon s}}{s^{2}+\mathrm{\varepsilon s}+{\hat{\omega }}^{2}}$$

**Figure 7:**
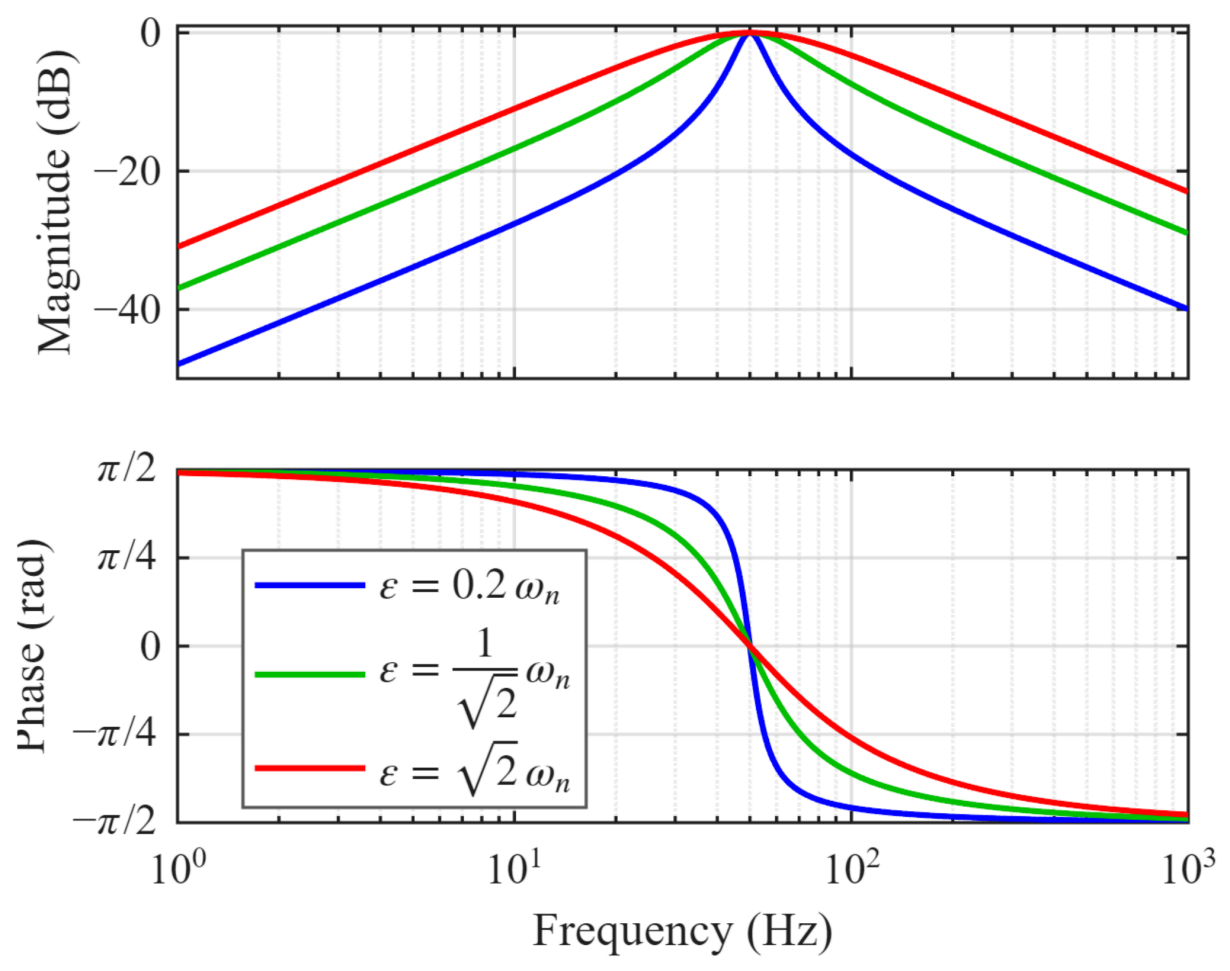
Bode plot of SFTF for different gains of *ε*.

From (22), it can be understood that the SFTF is actually a bandpass filter. In order to better visualize this case, the Bode plot of (22) for different gains of *ε* is demonstrated in Figure 7, where $\hat{\omega }$ is set to 2*π*50 rad/s. From this figure, the following conclusions about SFTF can be drawn.

(1) The SFTF provides a unity gain and zero phase at the fundamental frequency, i.e., 50 Hz. It also provides negative gain at non-fundamental frequencies due to its bandpass filter feature. This means that it significantly suppresses unwanted components.
(2) Its bandwidth increases for a larger *ε*, resulting in lower filtering capability.
(3) Its filtering capability improves with a lower *ε*; however, a lower *ε* leads to the dynamic response slowing down.

To ensure a trade-off between the filtering capability and dynamic response, *ε* is selected as $\left(1/\sqrt{2}\right)\omega_{n}$ (*ω_n_* is the fundamental frequency).

### 3.2. APF Structure

A quadrature-phase version of the input signal is required for operating the FLL mechanism. In standard SOGI-FLL, the SOGI structure provides this information (i.e., ${\hat{v}}_{\beta }$), as illustrated in Figure 1. On the contrary, the SFTF in the proposed method only gives the in-phase filtered form of the input signal *v*. Therefore, to obtain the signal ${\hat{v}}_{\beta }$, a quadrature-signal generator (QSG) is needed. For this purpose, the suggested FLL employs an all-pass filter-based QSG (APF-QSG) for integration with the SFTF, as shown in Figure 5.

The APF-QSG is extensively used owing to many advantages such as its simple structure, ease of implementation, fast dynamic response, etc. [36,37]. The transfer function of APF can be expressed in the *s*-domain as:

(23) $$G_{\mathrm{APF}}(s)=\frac{\hat{\omega }-s}{\hat{\omega }+s}$$

where $\hat{\omega }$ is the estimated frequency of the input signal. Substituting *s* = *jω* into (23), its magnitude and phase responses can be obtained as [38]:

(24) $$G_{\mathrm{APF}}(\mathrm{j\omega})=1∠-2\tan^{-1}\left(\frac{\omega }{\hat{\omega }}\right)$$

**Figure 8:**
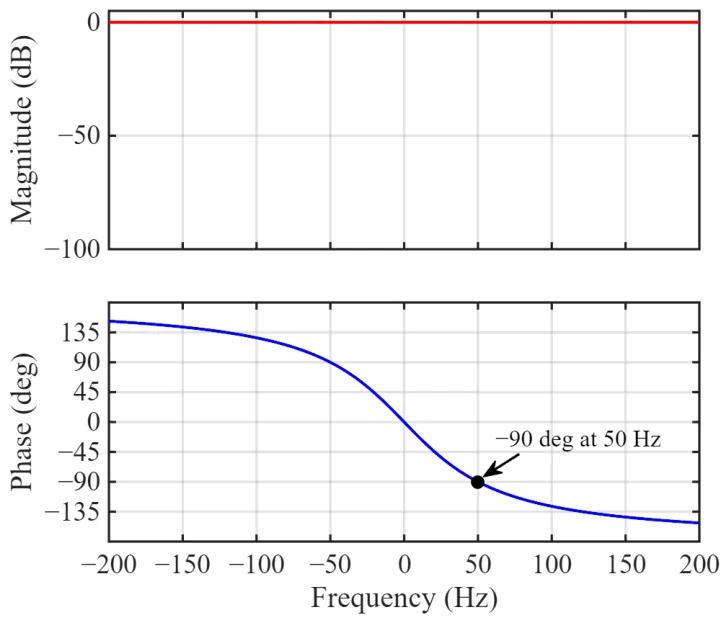
Bode plot of APF.

Figure 8 depicts the Bode diagram of APF. From (24) and Figure 8, the APF ensures unity gain for all frequencies, i.e., it does not affect the input signal magnitude, which means that it has no filtering capability. The APF creates a delay on the input signal phase. For example, if the estimated frequency $\hat{\omega }$ is equal to the fundamental frequency *ω* = 2*π*50 rad/s, a 90° phase delay occurs on the input signal.

From (23), the block structure of APF can be derived as shown in Figure 5. As observed, it requires only one multiplication, one integrator, and two subtractions, making it easy to apply to a system. The signal ${\hat{v}}_{\alpha }$ at the output of the SFTF is applied as an input for the APF, and the signal ${\hat{v}}_{\beta }$ is obtained from the APF, which is a quadrature-phase version of ${\hat{v}}_{\alpha }$. Using (22) and (23), and neglecting the FLL dynamics, the transfer functions of SFTF-FLL can be expressed in the *s*-domain as:

(25) $$G_{\alpha }(s)=\frac{{\hat{v}}_{\alpha }(s)}{v(s)}=\frac{\mathrm{\varepsilon s}}{s^{2}+\mathrm{\varepsilon s}+{\hat{\omega }}^{2}}$$

(26) $$G_{\beta }(s)=\frac{{\hat{v}}_{\beta }(s)}{v(s)}=\frac{\mathrm{\varepsilon s}\left(\hat{\omega }-s\right)}{\left(\hat{\omega }+s\right)\left(s^{2}+\mathrm{\varepsilon s}+{\hat{\omega }}^{2}\right)}$$

**Figure 9:**
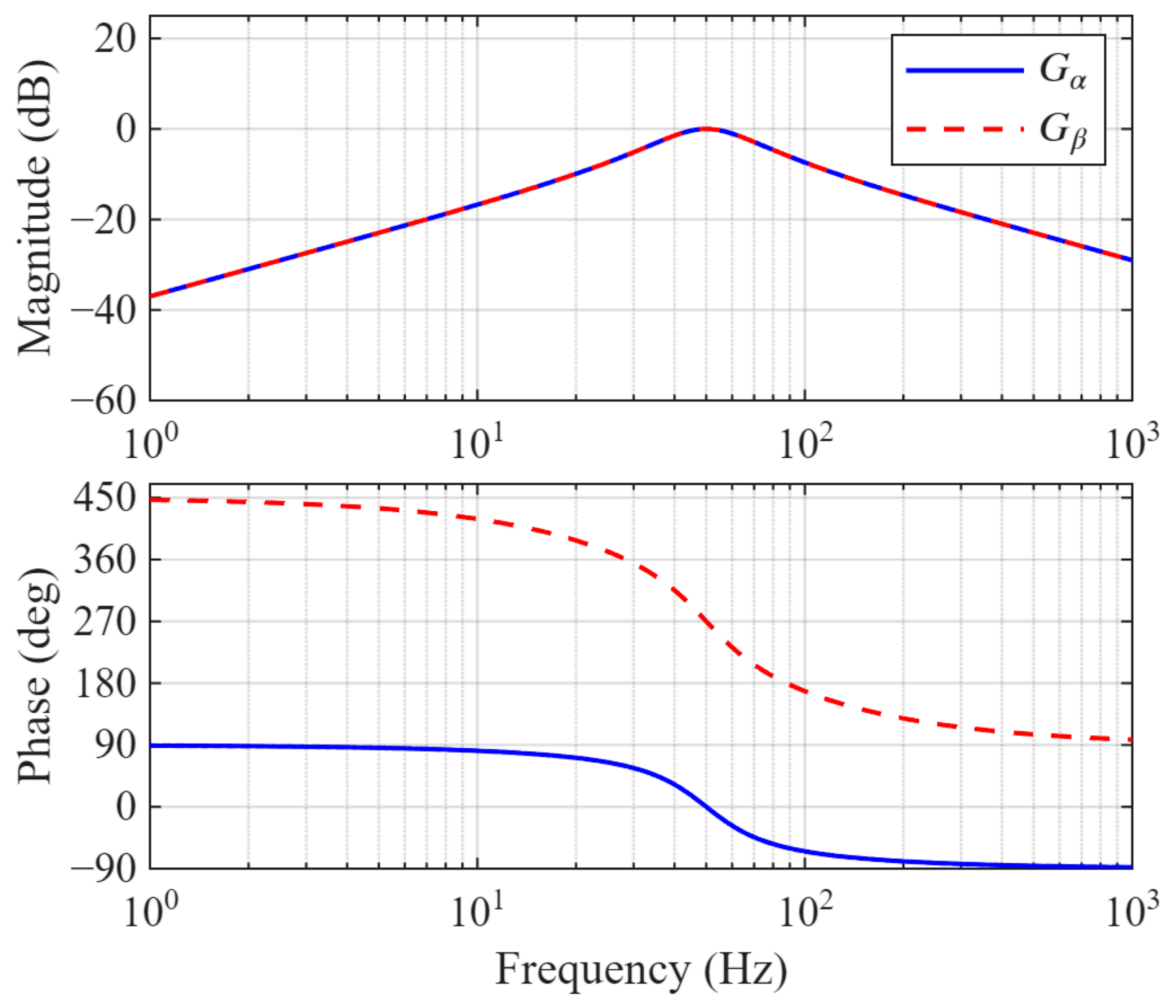
Bode plots of *G_α_*(*s*) and *G_β_*(*s*) for SFTF-FLL.

Using (25) and (26), the Bode diagrams of the transfer functions *G_α_*(*s*) and *G_β_*(*s*) are obtained as in Figure 9, where $\varepsilon =(1/\sqrt{2})\omega_{n}$ and $\hat{\omega }=2\pi 50$ rad/s. As observed, unlike the standard SOGI-FLL, both *G_α_*(*s*) and *G_β_*(*s*) act as bandpass filters. Please note that, as shown earlier in Figure 2, *G_β_*(*s*) of SOGI-FLL exhibits low-pass filter characteristics. As demonstrated in Figure 9, the SFTF-FLL exhibits better filtering performance compared to the SOGI-FLL, thanks to the bandpass filter characteristics of the SFTF-FLL’s *G_β_*(*s*).

Finally, ${\hat{v}}_{\beta }$ at the APF output is transferred into the FLL structure to extract the input signal frequency. FLL structure of the proposed technique, as shown in Figure 5, is identical to that of the SOGI-FLL. According to *ε*, the FLL gain *λ* will be determined in Section 3.3.

### 3.3. Small-Signal Model and Parameter Design of the Proposed SFTF-FLL

**Figure 10:**
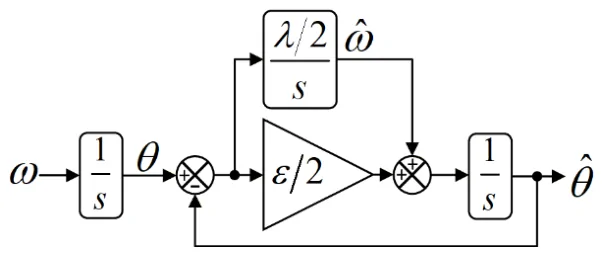
Small-signal model of the SFTF-FLL.

The small-signal model of the standard SOGI-FLL has been derived in Section 2, which facilitates the derivation of the small-signal model for the proposed SFTF-FLL. To conserve space and avoid redundancy, the small-signal modeling procedure of SFTF-FLL is not described again, as it is identical to that of SOGI-FLL. Based on (3)–(13) and Figure 5, its small-signal model can be easily derived, as given in Figure 10.

To verify the accuracy of the model, two simulation studies are carried out as follows.

Test-1: A 20° phase jump is created.

Test-2: A +2 Hz frequency jump occurs.

**Figure 11:**
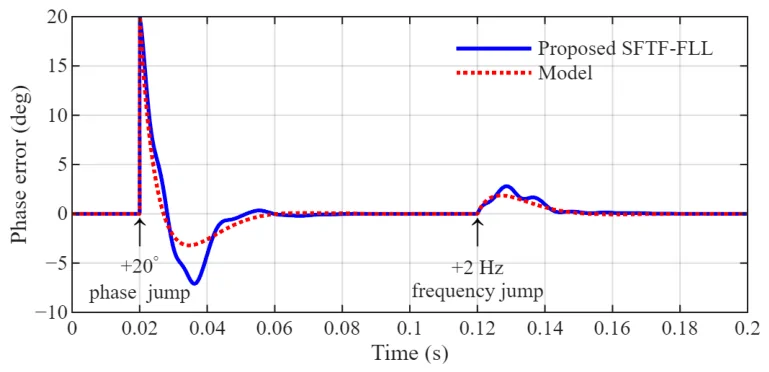
Accuracy evaluation of the small-signal model of SFTF-FLL.

In these tests, the estimated phase error by the SFTF-FLL in Figure 5 is compared with that of its small-signal model in Figure 10. The results are provided in Figure 11. As can be seen, the small-signal model predicts the average dynamic behavior of SFTF-FLL with sufficient accuracy.

From the small-signal model in Figure 10, the open-loop transfer function of the SFTF-FLL can be obtained as:

(27) $$G_{\mathrm{ol}}(s)=\frac{1}{2}\underset{\mathrm{quasi}\;\mathrm{PI}\text{-}\mathrm{type}\;\mathrm{controller}}{\underset{︸}{\left(\varepsilon +\frac{\lambda }{s}\right)}}\underset{\mathrm{VCO}}{\underset{︸}{\frac{1}{s}}}$$

where *ε* and *λ* correspond to proportional gain and integral gain, respectively. VCO is a voltage-controlled oscillator.

Please note that the small-signal model of the SFTF-FLL illustrated in Figure 10 is similar to that of SOGI-FLL illustrated in Figure 3. Thus, based on (14)–(19), the FLL gain *λ* can be determined as:

(28) $$\lambda =\frac{\varepsilon^{2}}{8\xi^{2}}$$

Using (28), if the SFTF gain *ε* is considered to be $(1/\sqrt{2})\omega_{n}$, discussed in Section 3.1 and Figure 7, and the damping factor *ξ* is taken into consideration as $1/\sqrt{2}$, *λ* = 12,337 is obtained.

Table 2 quantitatively confirms the frequency-domain observations presented in Figure 7. The SFTF-FLL is evaluated for three different values of *ε*, while the corresponding FLL gain *λ* is recalculated using the proposed small-signal-based tuning procedure described in (28). Increasing *ε* from 0.2*ω_n_* to $\sqrt{2}\omega_{n}$ reduces the phase settling time from 220 ms to 40 ms. However, the frequency overshoot increases from 1.13 Hz to 10.3 Hz, while the peak-to-peak phase errors under harmonic distortion, sensor-induced DC-offset, and combined sensor-induced DC-offset and noise conditions increase from 0.72°, 0.12°, and 1.6° to 6.95°, 7.43°, and 13°, respectively. Thus, a large value of *ε* improves transient response but reduces filtering capability. A small value *ε*, on the other hand, provides stronger filtering but results in a significantly slower dynamic response. Accordingly, the intermediate value is selected to obtain a reasonable trade-off between dynamic response and filtering capability.

## 4. Numerical Results

**Figure 12:**
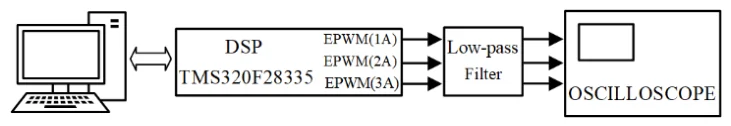
Experimental setup.

This section evaluates the performance of the proposed SFTF-FLL via some experimental studies. Figure 12 illustrates the experimental setup. This setup consists of a personal computer, a TMS320F28335 DSP (Texas Instruments, Dallas, TX, USA), an external low-pass filter, and a digital oscilloscope. In the experiments, for the sake of simplicity, a single-phase input signal is generated virtually, and the proposed method and the other FLL methods used in comparison are implemented on the DSP development board. In other words, experimental validation is performed using a real-time processor-in-the-loop (PIL) configuration on the TMS320F28335 floating-point DSP operating at a 10 kHz sampling frequency. In the literature, the PIL test is a widely accepted and well-established verification method specifically designed to verify complex control and synchronization algorithms. The PIL also provides a precise and repeatable environment to evaluate the core computational complexity and digital filtering robustness of the developed method [39,40,41]. To obtain the graphs of estimated amplitude, estimated frequency, and phase error of the input signal from the enhanced pulse width modulator (EPWM) of the TMS320F28335 DSP, these signals are fed into the low-pass filter. During the experiments, the amplitude of the input signal and the sampling frequency are adjusted to 1 p.u. and 10 kHz, respectively.

**Figure 13:**
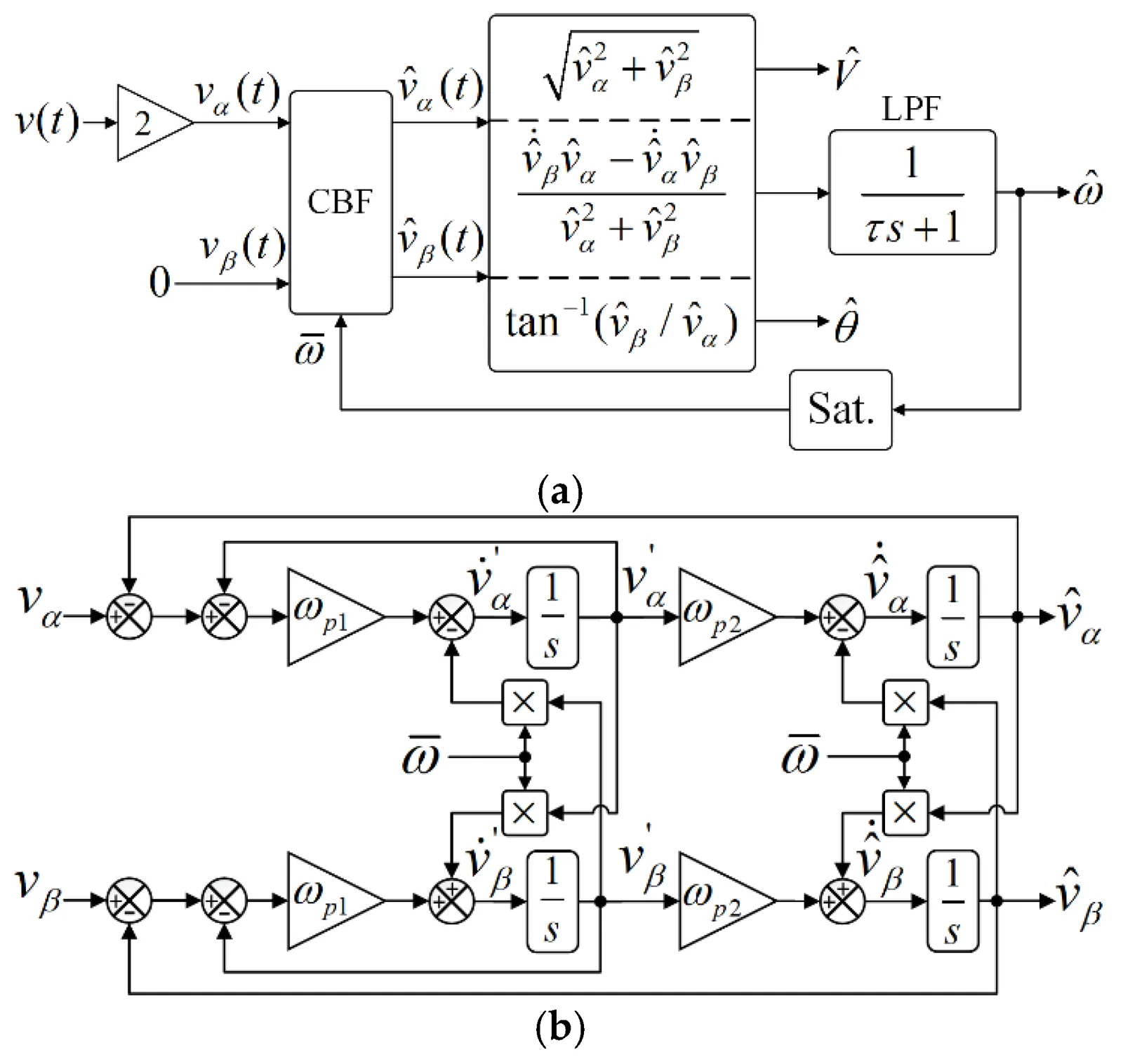
Block structure of CBF-FLL: (**a**) its general block representation and (**b**) block structure of a CBF.

To validate the effectiveness of the proposed SFTF-FLL method, the results of the proposed method shown in Figure 5 are compared with those of the standard SOGI-FLL [21] presented earlier in Figure 1 and those of the complex bandpass filter-based FLL (CBF-FLL) [20] shown in Figure 13. The SOGI-FLL and CBF-FLL are selected as the primary experimental benchmarks for specific reasons. The SOGI-FLL represents the conventional and widely used single-phase FLL structure and employs a frequency-adaptation mechanism comparable to that of the proposed method. Hence, it enables the effect of the proposed SFTF–APF signal-processing stage to be evaluated directly. The CBF-FLL, on the other hand, is an advanced, competitive, and non-SOGI single-phase FLL with bandpass filtering capability. Consequently, the SOGI-FLL and CBF-FLL are chosen to compare the performance of the proposed SFTF-FLL.

The parameters of all methods are provided in Table 3. The following tests are created.

Test Scenario-1: A +40° phase jump happens in the input signal.

Test Scenario-2: The dominant low-order harmonics, i.e., 3rd, 5th, 7th, 9th, and 11th are injected to input signal. Additionally, sub-and inter-harmonics are added to the input signal to create a more dynamic harmonic profile. The frequency, magnitude, and phase angle of each injected component are listed in Table 4, resulting in an input-voltage total harmonic distortion (THD) of 8.43%. In this test, to evaluate the filtering capability of FLLs in the case of frequency change, the input signal frequency is also jumped from 50 Hz to 52 Hz, which is the maximum frequency limit specified in the EN50160 standard [42].

Test Scenario-3: A constant offset of 0.1 p.u. is digitally added to the measured voltage signal to emulate a severe sensor-induced DC offset. Therefore, the test evaluates the immunity of the FLL methods against measurement errors. The DC-offset magnitude of 0.1 p.u. is specifically selected as a worst-case benchmark to rigorously evaluate the sensor-induced DC-offset rejection capabilities of the FLL methods under severe sensor measurement errors (e.g., analog-to-digital converter (ADC) offset drifts and operational amplifier offsets).

Test Scenario-4: To evaluate the performance and robustness of the FLL algorithms under more realistic measurement conditions, a compound disturbance scenario incorporating both sensor-induced DC offset and measurement noise is created. In this scenario, a band-limited white Gaussian noise with a signal-to-noise ratio (SNR) of 17 dB is superimposed onto an input signal containing a 0.1 p.u. DC offset. This test mimics the high-frequency measurement noise, operational amplifier thermal fluctuations, and ADC quantization interference typically introduced by physical analog sensor conditioning circuits.

**Figure 14:**
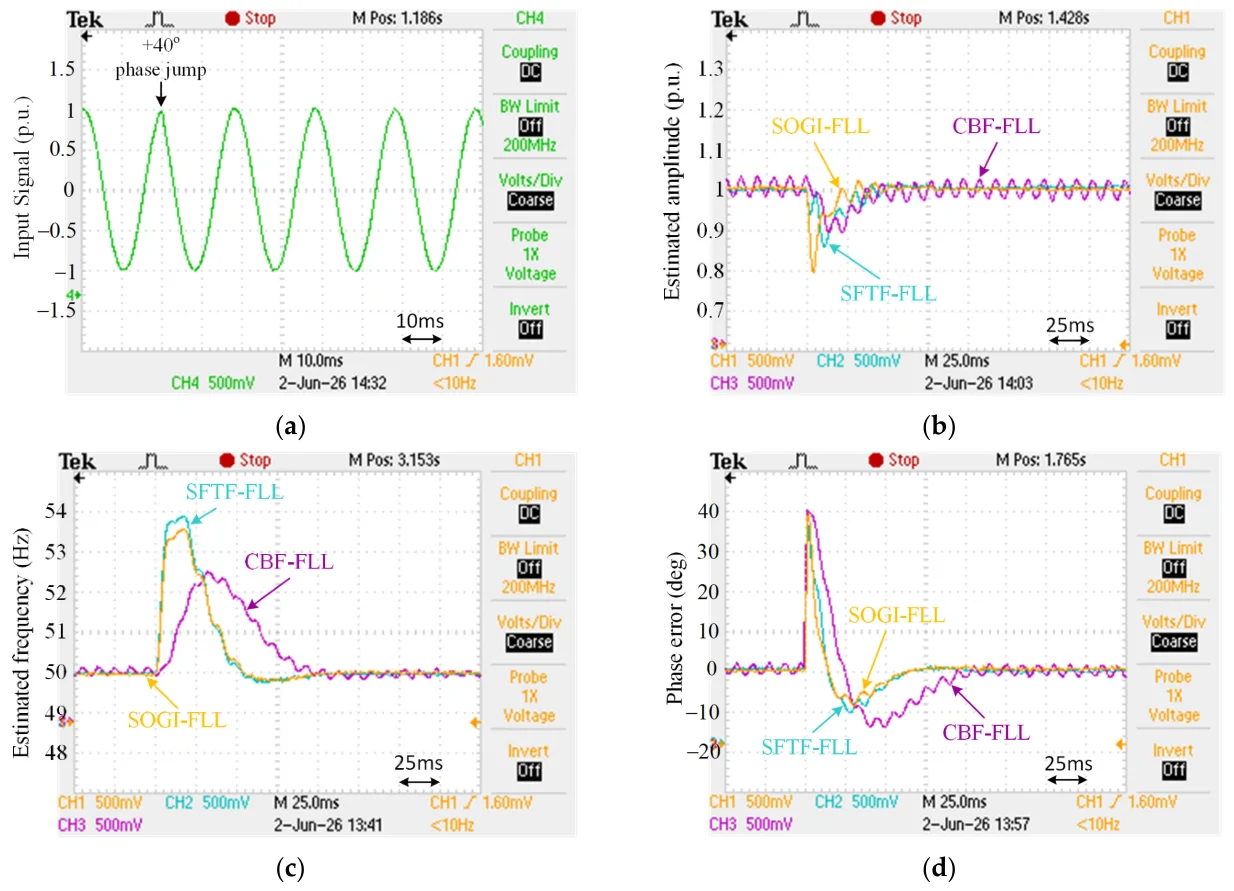
Experimental waveforms for Test Scenario-1 in the case of a +40° phase jump. (**a**) Input signal, (**b**) estimated amplitude, (**c**) estimated frequency, and (**d**) Phase error.

Figure 14 demonstrates the experimental results of Test Scenario-1 under the +40° phase jump condition. As shown, among the three FLL methods, the CBF-FLL takes the longest convergence time and fails to reach stability according to the 2% stability criterion. Additionally, it leads to double-frequency oscillation errors when estimating the amplitude, frequency, and phase of the input signal, which means it is not suitable for grid applications. Detailed numerical findings are provided in Table 5. On the other hand, the SOGI-FLL and proposed SFTF-FLL exhibit similar dynamic performance. As can be seen in Table 2, their convergence times are 62.3 ms and 61 ms, respectively. Moreover, they have no steady-state errors in the estimation of the input signal information. However, their frequency overshoots are slightly greater than those of the CBF-FLL. This overshoot can be significantly reduced using the adaptive mechanism introduced in [43]. As a result, considering Figure 14 and the numerical values in Table 5, while the proposed SFTF-FLL and SOGI-FLL methods exhibit similar performance, they perform better than the CBF-FLL in the case of a phase jump.

**Figure 15:**
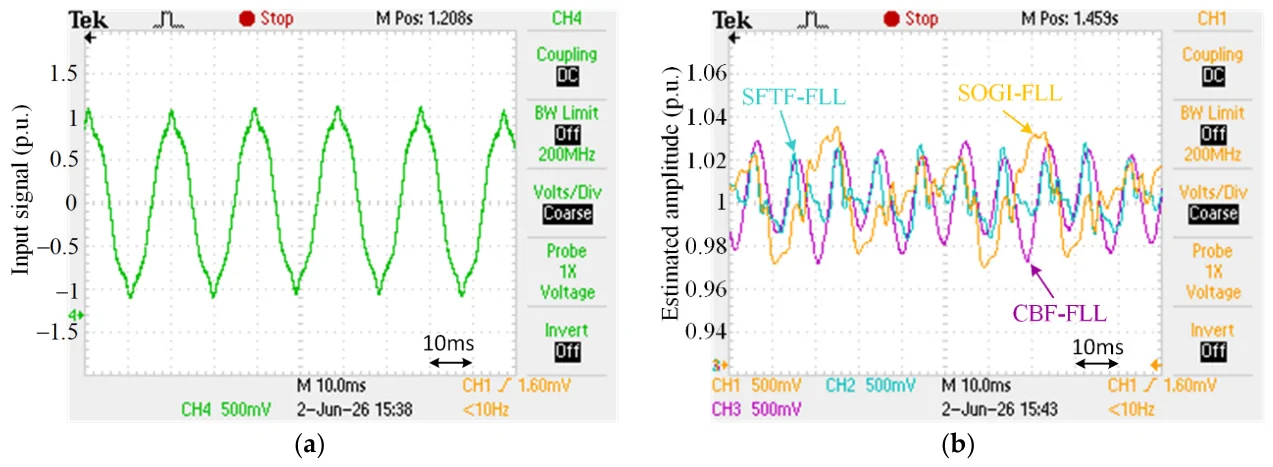

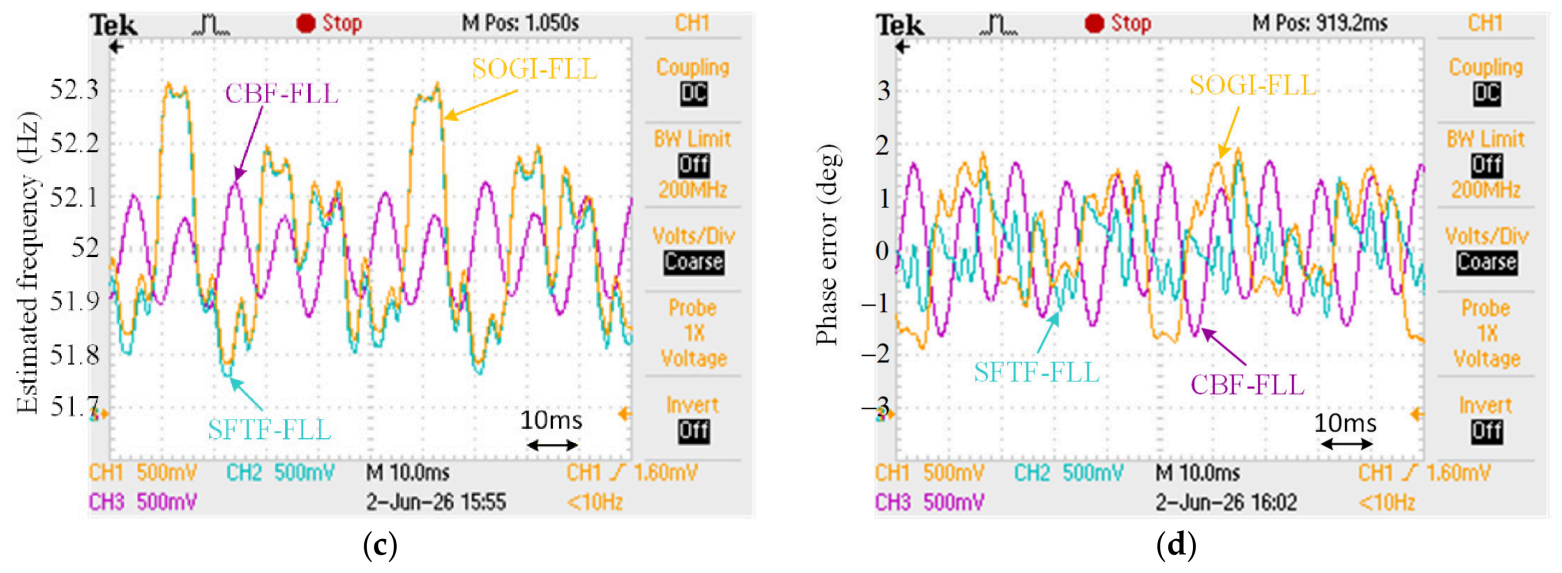
Experimental waveforms for Test Scenario-2 in the presence of various harmonic components in the input signal. (**a**) Input signal, (**b**) Estimated amplitude, (**c**) Estimated frequency, and (**d**) Phase error.

**Figure 16:**
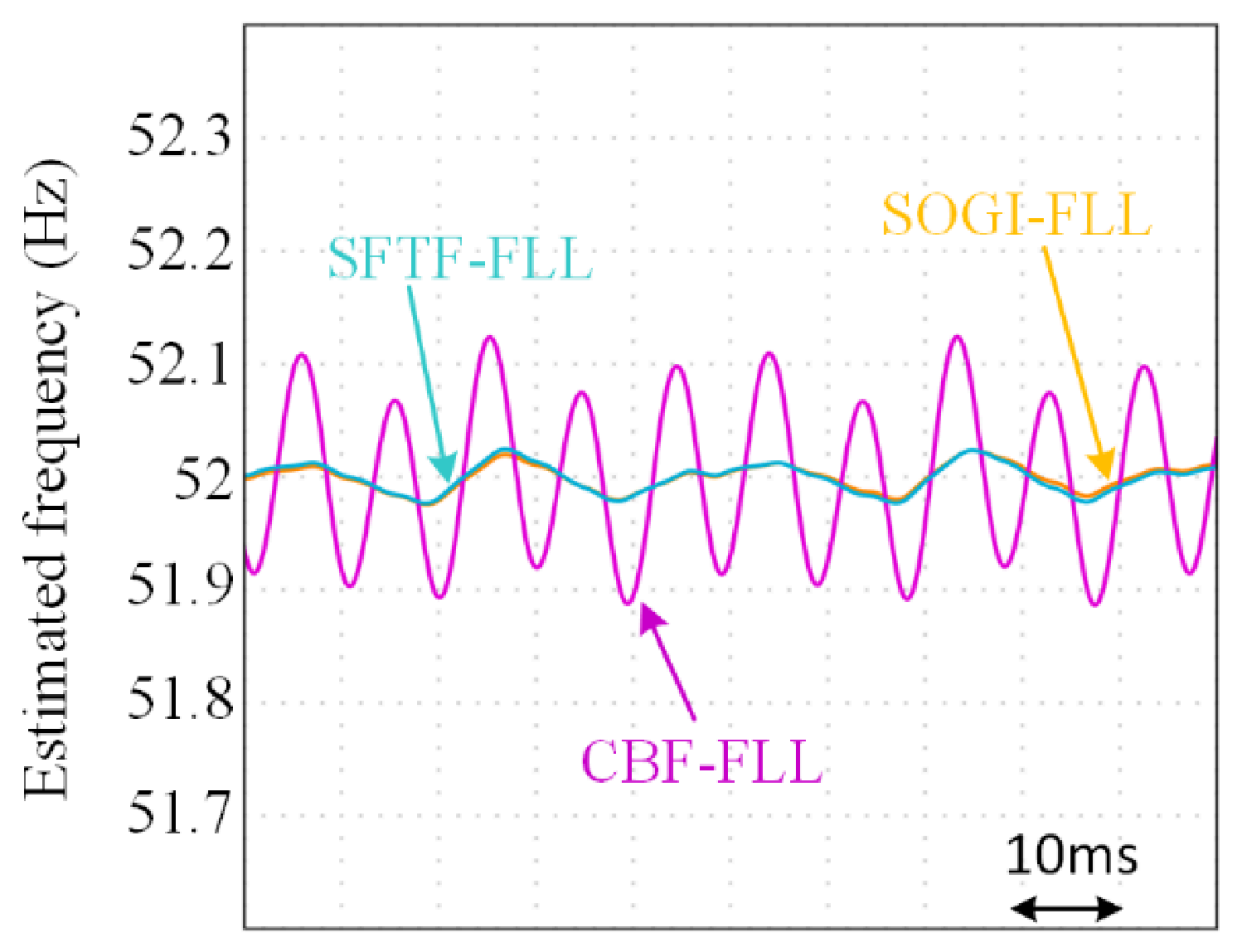
Estimated frequency responses under equivalent low-pass-filtering condition for Test Scenario-2.

Figure 15 depicts the experimental results of Test Scenario-2 under a harmonically distorted input signal with a +2 Hz frequency jump. As observed, all FLLs provide a similar level of accuracy in steady-state. However, as can be seen in Table 5, while the peak-to-peak amplitude errors of SOGI-FLL and CBF-FLL are 0.066 p.u. and 0.054 p.u., respectively, this error is 0.047 p.u. for SFTF-FLL. Furthermore, the proposed SFTF-FLL, which has a 3° phase error in steady-state, offers a lower peak-to-peak phase error than the other two FLLs. The peak-to-peak phase errors in SOGI-FLL and CBF-FLL are 4° and 3.3°, respectively. On the other hand, as observed in Figure 15c, the CBF-FLL results in the lowest frequency error. The reason for this is that the frequency component is passed through an additional LPF, as shown in the structure of CBF-FLL in Figure 13a. If the frequency signals in SOGI-FLL and SFTF-FLL are also passed through an LPF, their frequency errors can be significantly reduced. To verify this case and ensure a fairer comparison of frequency errors, the LPF is added to the frequency output of SOGI-FLL and SFTF-FLL, just as in CBF-FLL. Figure 16 presents the resulting frequency estimates under harmonically distorted conditions. As can be seen, the additional filtering on the frequency path significantly reduces the frequency fluctuations of the SOGI-FLL and SFTF-FLL. However, the additional LPF slows down the FLL transient response. For this reason, a trade-off must be made between the smoothness of the frequency response and the FLL response speed.

**Figure 17:**
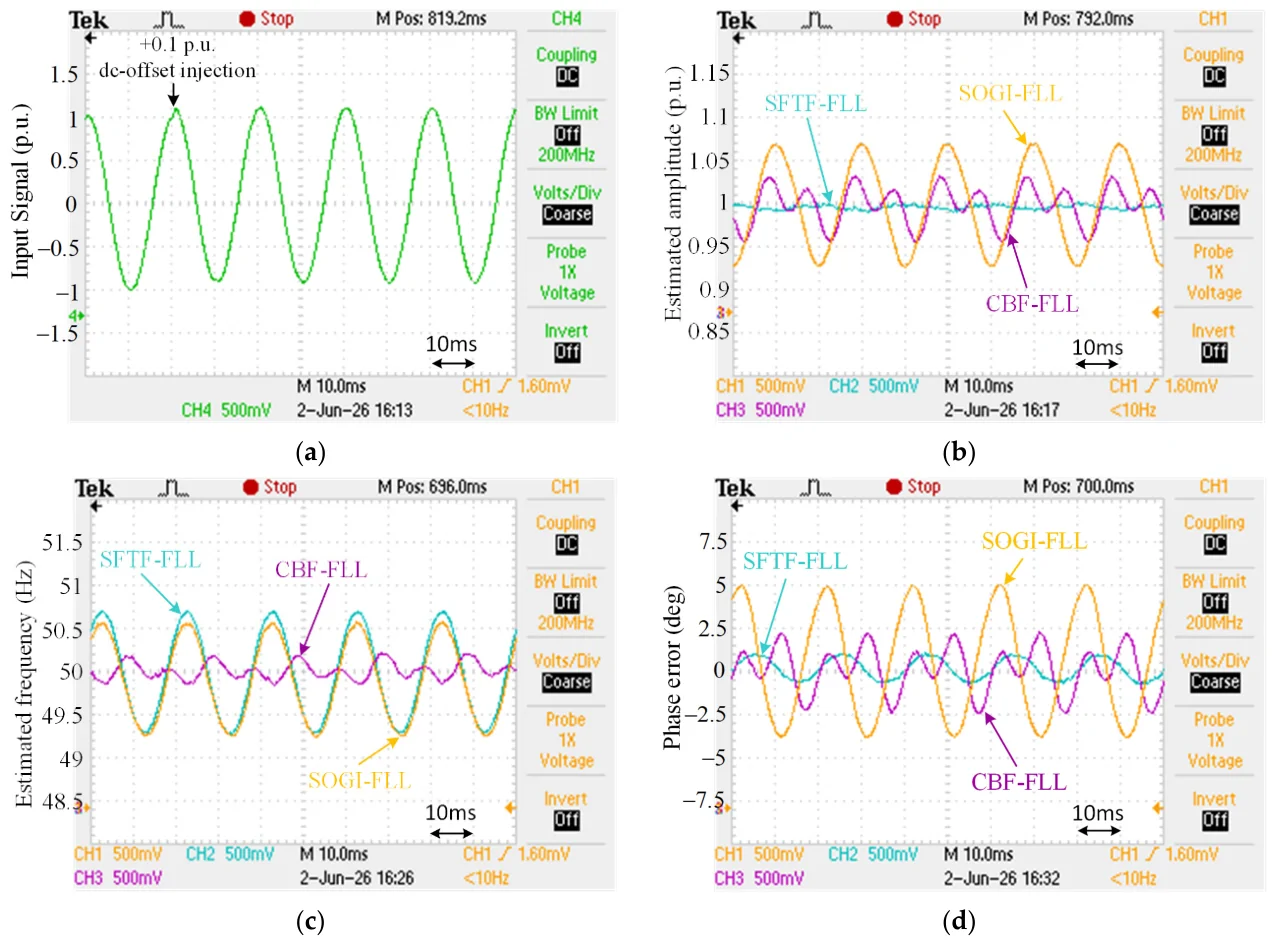
Experimental waveforms for Test Scenario-3 in the presence of a sensor-induced DC-offset component in the input signal. (**a**) Input signal, (**b**) Estimated amplitude, (**c**) Estimated frequency, and (**d**) Phase error.

**Figure 18:**
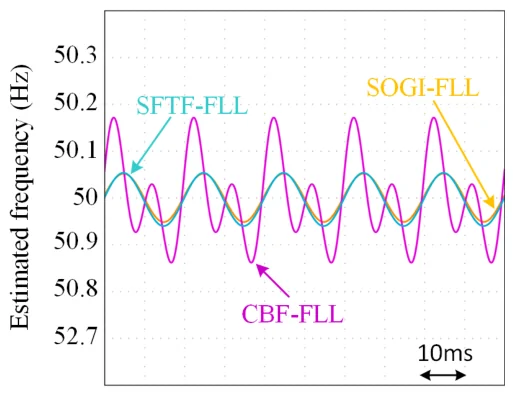
Estimated frequency responses under equivalent low-pass-filtering condition for Test Scenario-3.

Figure 17 provides the experimental results of Test Scenario-3 in the case of sensor-induced DC offset. As shown in Figure 17b, the SOGI-FLL and CBF-FLL cause significant amplitude errors. As given in Table 5, their peak-to-peak amplitude errors are 0.14 p.u. and 0.074 p.u., respectively; in contrast, the peak-to-peak amplitude error in the proposed FLL is nearly zero (0.007 p.u.). The reason behind this, as discussed in Section 3.2, is that the transfer functions of the proposed method—i.e., both *G_α_*(*s*) and *G_β_*(*s*)—exhibit bandpass filter characteristics. On the other hand, the CBF-FLL achieves a lower frequency error thanks to the additional LPF in its structure, as illustrated in Figure 17c. Its peak-to-peak frequency error is 0.31 Hz, which is lower than the 1.28 Hz and 1.36 Hz obtained with the SOGI-FLL and SFTF-FLL, respectively. Nevertheless, their frequency errors can be significantly reduced by placing an LPF on the frequency paths. The results are provided in Figure 18. From Figure 17d, the peak-to-peak phase error of the suggested SFTF-FLL is considerably smaller than that of SOGI-FLL and CBF-FLL, indicating that the proposed method provides a significant improvement against the sensor-induced DC offsets. Detailed numerical results are presented in Table 5.

**Figure 19:**
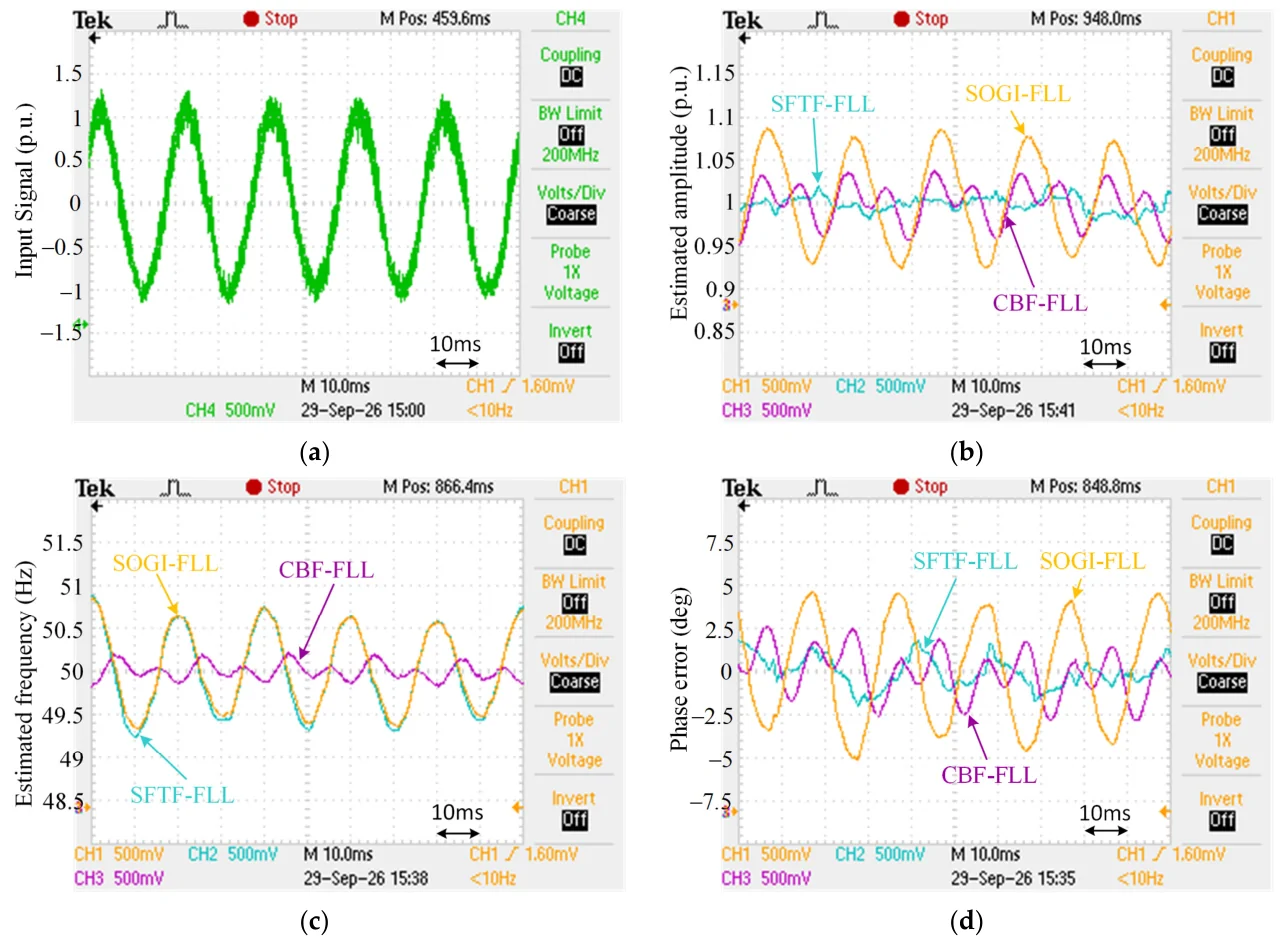
Experimental waveforms for Test Scenario-4 in the presence of a sensor-induced DC-offset component and measurement noise in the input signal. (**a**) Input signal, (**b**) Estimated amplitude, (**c**) Estimated frequency, and (**d**) Phase error.

Figure 19 presents the experimental results of Test Scenario-4 under the combined presence of sensor-induced DC offset and measurement noise. As shown in Figure 19a, the input signal is visibly distorted by measurement noise. Figure 19b shows that the estimated amplitude of the proposed SFTF-FLL exhibits considerably smaller oscillations than those of the SOGI-FLL and CBF-FLL. Although the additional LPF in the CBF-FLL structure provides a smoother frequency estimate, as illustrated in Figure 19c, the proposed method provides more accurate amplitude and phase estimates. In particular, Figure 19d shows that the phase error oscillations of the SFTF-FLL are significantly smaller than those of the other methods. These results demonstrate that the bandpass characteristics of both the in-phase and quadrature-phase signals in the proposed structure improve its robustness against the simultaneous presence of sensor-induced DC offset and measurement noise. Detailed numerical results are provided in Table 5.

To further evaluate the robustness of the algorithms against varying levels of the sensor-induced DC-offset disturbances, a quantitative sensitivity study is conducted across four distinct DC-offset levels: low (0.02 p.u.), medium (0.05 p.u.), high (0.1 p.u.), and extreme (0.2 p.u.). Please note that throughout the tests, the frequency signal is passed through the LPF in each FLL method. The peak-to-peak amplitude, frequency, and phase errors for SOGI-FLL, CBF-FLL, and the proposed SFTF-FLL under these conditions are detailed in Table 6. As presented in Table 6, as the sensor-induced DC offset increases from 0.02 p.u. to 0.2 p.u., the estimation errors for all methods increase. Specifically, the peak-to-peak amplitude error of SOGI-FLL increases from 0.028 p.u. to 0.28 p.u., and its phase error expands from 1.6° to 16°. The CBF-FLL exhibits a similar trend, with its amplitude error increasing from 0.053 p.u. to 0.1 p.u. and phase error rising from 3.06° to 6.1°. In contrast, the proposed SFTF-FLL demonstrates remarkable DC-offset rejection capabilities across all tested levels. Even under an extreme condition of 0.2 p.u. DC offset, SFTF-FLL maintains a peak-to-peak amplitude error of only 0.03 p.u. and a phase error of 3°. Furthermore, the peak-to-peak frequency error of SFTF-FLL remains low (ranging between 0.023 Hz and 0.23 Hz), which is close to SOGI-FLL (0.021 Hz to 0.21 Hz) and significantly better than CBF-FLL (0.22 Hz to 0.44 Hz). The proposed SFTF-FLL achieves this robustness because the in-phase and quadrature-phase components of its input signal exhibit the characteristics of a bandpass filter (as discussed in Section 3.2 and shown in Figure 9), which effectively suppress the sensor-induced DC offsets.

To evaluate both transient response speed and long-term tracking accuracy under a unified criterion, the integral of time-weighted absolute error (ITAE) index is calculated for amplitude, frequency, and phase estimates across all test scenarios, defined as:

(29) $$\mathrm{ITAE}=\int_{0}^{T}t\left|e(t)\right|\mathrm{dt}$$

where |*e*(*t*)| denotes the absolute error and *T* is the total evaluation interval.

Because the ITAE index heavily penalizes errors that persist over time, it provides a comprehensive measure of dynamic convergence and steady-state suppression capability. The quantitative ITAE results are summarized in Table 7.

Table 7 reveals that no single method provides the minimum ITAE for every estimated quantity and operating condition. Under the phase jump condition (Case-1), the SOGI-FLL achieves the lowest amplitude and phase ITAE, whereas the SFTF-FLL provides the lowest frequency ITAE. Under harmonic distortion (Case-2), DC offset (Case-3), and measurement noise (Case-4) conditions, the SFTF-FLL achieves the lowest amplitude and phase ITAE values, confirming its enhanced disturbance rejection capability in these estimated quantities. However, CBF-FLL provides the lowest frequency ITAE under these conditions. Overall, the proposed SFTF-FLL offers a favorable compromise, particularly in amplitude and phase estimation under distorted, DC-offset, and noisy conditions, rather than universal superiority across all performance indices.

## 5. Conclusions

In this paper, a non-SOGI single-phase SFTF-based FLL, referred to as SFTF-FLL, has been proposed. The small-signal model was derived to perform the parameter design of the suggested method. To prove the effectiveness of the suggested SFTF-FLL method, the experimental results of the suggested method were compared with those of the standard SOGI-FLL and CBF-FLL. The numerical results obtained show that the proposed FLL estimates the amplitude and phase of the input signal more accurately in the presence of harmonics, sensor-induced DC offset, and measurement noise, even though the CBF-FLL determines the frequency more accurately thanks to its LPF. In particular, the proposed SFTF-FLL provides better performance in suppressing sensor-induced DC-offset errors, resulting in more accurate amplitude and phase estimation. Considering this fact, it can be concluded that the proposed SFTF-FLL is an alternative for applications that prioritize amplitude and phase accuracy.

## Figures and Tables

**Table 1:** Key features of FLL methods.

Method	Fundamental Extraction	Quadrature Generation	Difference from Proposed Method
Standard SOGI-FLL	SOGI resonant structure	SOGI quadrature output	Quadrature channel has an LPF characteristic
Prefiltered/in-loop-filtered SOGI-FLL	SOGI with additional filtering	SOGI quadrature output	Retains the SOGI core and includes additional SOGI
MSOGI-FLL	Parallel harmonic-selective SOGIs	SOGI quadrature output	Requires additional SOGI units for selected harmonic orders
CBF-FLL	Complex bandpass filtering	Complex orthogonal representation	Uses a complex signal representation and sequence separation
Transfer-delay FLL	Delayed signal cancellation operator	Time-delayed signal	Frequency deviations produce quadrature errors
Comb-filter FLL	Delayed-based filtering	SOGI quadrature output	Requires delay memory and is sensitive to frequency deviation
Proposed FLL	SFTF structure	Frequency-adaptive APF applied to SFTF output	

**Table 2:** Effect of the SFTF gain on the dynamic response disturbance sensitivity of the SFTF-FLL.

SFTF Gain (*ε*)	FLL Gain (*λ*)	Phase Settling Time	Frequency Overshoot	Phase Error Under Harmonic Distortion	Phase Error Under Sensor-Induced DC Offset	Phase Error Under Sensor-Induced DC Offset and Noise
0.2*ω_n_*	987	220 ms	1.13 Hz	0.72°	0.12°	1.6°
$(1/\sqrt{2})\omega_{n}$	12,337	61 ms	3.94 Hz	3°	1.5°	4.5°
$\sqrt{2}\omega_{n}$	49,348	40 ms	10.3 Hz	6.95°	7.43°	13°

The evaluated conditions correspond to the phase jump, harmonic distortion, sensor-induced DC offset, and measurement noise scenarios described later in Section 4. In this table, the phase error values represent the peak-to-peak steady-state errors and *ω_n_* = 2*π*50 rad/s.

**Table 3:** Control Parameters of FLLs.

Method	Parameters
SOGI-FLL [21]	$k=1/\sqrt{2}$, *λ* = 12,337
CBF-FLL [20]	*ω_p_*_1_ = 151.7, *ω_p_*_2_ = 62.8, *τ* = 0.0384
SFTF-FLL	$\varepsilon =(1/\sqrt{2})\omega_{n}=222.14$, *λ* = 12,337

**Table 4:** Composition of the distorted input signal used in the harmonic test.

Component	Frequency (Hz)	Magnitude (p.u.)	Phase Angle (°)
Fundamental	50	1	0
3rd harmonic	150	0.04	0
5th harmonic	250	0.05	0
7th harmonic	350	0.04	0
9th harmonic	450	0.01	0
11th harmonic	550	0.03	0
Sub-harmonic	10	0.04	0
Inter-harmonic	205	0.02	0

**Table 5:** Experimental results of FLLs.

	SOGI-FLL	CBF-FLL	SFTF-FLL
Test Scenario-1: Phase jump			
Phase settling time	62.3 ms (3.12 cycles)	-	61 ms (3.05 cycles)
Peak amplitude deviation	0.2 p.u.	0.1 p.u.	0.14 p.u.
Frequency overshoot	3.54 Hz	2.5 Hz	3.94 Hz
Phase overshoot	9°	14.6°	11°
Peak-to-peak amplitude error in steady-state	0 p.u.	0.048 p.u.	0 p.u.
Peak-to-peak frequency error in steady-state	0 Hz	0.2 Hz	0 Hz
Peak-to-peak phase error in steady-state	0°	2.75°	0°
Test Scenario-2: Harmonics			
Peak-to-peak amplitude error	0.066 p.u.	0.054 p.u.	0.047 p.u.
Peak-to-peak frequency error	0.54 Hz	0.24 Hz	0.55 Hz
Peak-to-peak phase error	4°	3.3°	3°
Test Scenario-3: Sensor-induced DC offset			
Peak-to-peak amplitude error	0.14 p.u.	0.074 p.u.	0.007 p.u.
Peak-to-peak frequency error	1.28 Hz	0.31 Hz	1.36 Hz
Peak-to-peak phase error	8.5°	4.4°	1.5°
Test Scenario-4: Sensor-induced DC offset and measurement noise			
Peak-to-peak amplitude error	0.17 p.u.	0.1 p.u.	0.007 p.u.
Peak-to-peak frequency error	1.53 Hz	0.45 Hz	1.63 Hz
Peak-to-peak phase error	10°	6.5°	4.7°

**Table 6:** Quantitative sensitivity analysis of FLL methods under various sensor-induced DC-offset levels.

DC-Offset Level	Method	Peak-to-Peak Amplitude Error	Peak-to-Peak Frequency Error	Peak-to-Peak Phase Error
0.02 p.u. (Low)	SOGI-FLL	0.028 p.u.	**0.021 Hz**	1.6°
	CBF-FLL	0.053 p.u.	0.22 Hz	3.06°
	SFTF-FLL	**0.003 p.u.**	0.023 Hz	**0.3°**
0.05 p.u. (Medium)	SOGI-FLL	0.071 p.u.	**0.052 Hz**	4°
	CBF-FLL	0.061 p.u.	0.25 Hz	3.5°
	SFTF-FLL	**0.007 p.u.**	0.057 Hz	**0.7°**
0.1 p.u. (High)	SOGI-FLL	0.142 p.u.	**0.1 Hz**	8°
	CBF-FLL	0.074 p.u.	0.31 Hz	4.4°
	SFTF-FLL	**0.015 p.u.**	0.11 Hz	**1.5°**
0.2 p.u. (Extreme)	SOGI-FLL	0.28 p.u.	**0.21 Hz**	16°
	CBF-FLL	0.1 p.u.	0.44 Hz	6.1°
	SFTF-FLL	**0.03 p.u.**	0.23 Hz	**3°**

Lower values indicate better performance, and the lowest values are shown in bold.

**Table 7:** Quantitative ITAE-based performance comparison.

Case	Method	Amplitude ITAE	Frequency ITAE	Phase ITAE
Case-1 Phase jump	SOGI-FLL	**0.00003160**	0.0027318	**0.0104265**
	CBF-FLL	0.00032671	0.0053664	0.0476861
	SFTF-FLL	0.00004794	**0.0025351**	0.0115768
Case-2 Harmonics	SOGI-FLL	0.00025995	0.0400833	0.251206
	CBF-FLL	0.00027249	**0.0381919**	0.535540
	SFTF-FLL	**0.00017960**	0.0394953	**0.210832**
Case-3 DC-offset	SOGI-FLL	0.00086444	0.0083143	0.0542342
	CBF-FLL	0.00035483	**0.0014724**	0.0204562
	SFTF-FLL	**0.00004249**	0.0087832	**0.0105131**
Case-4 DC-offset with noise	SOGI-FLL	0.00090915	0.00857804	0.05603590
	CBF-FLL	0.00037982	**0.00158773**	0.02223843
	SFTF-FLL	**0.00019719**	0.00903606	**0.01482362**

All indices were calculated over the same 200 ms interval following the application of the disruptive effect. Lower values indicate better performance, and the lowest values are shown in bold.

## Data Availability

The original contributions presented in this study are included in the article. Further inquiries can be directed to the corresponding author.

## Abbreviations

The following abbreviations are used in this manuscript:

SOGI	Second-order generalized integrator
FLL	Frequency-locked loop
SOGI-FLL	Second-order generalized integrator-frequency-locked loop
SFTF	Synchronous frequency tracking filter
SFTF-FLL	Synchronous frequency tracking filter-frequency-locked loop
CBF	Complex bandpass filter
CBF-FLL	Complex bandpass filter-frequency-locked loop
PWM	Pulse width modulation
SVPWM	Space vector pulse width modulation
SiC	Silicon carbide
PLL	Phase-locked loop
LPF	Low-pass filter
MSOGI-FLL	Multiple second-order generalized integrator-frequency-locked loop
LKF	Linear Kalman filter
TD-AFLL	Transfer-delay-adaptive-frequency-locked loop
ES-FLL	Extremum-seeking-frequency-locked loop
FOGI-FLL	Fourth-order generalized integrator-frequency-locked loop
APF	All-pass filter
LTI	Linear time-invariant
QSG	Quadrature-signal generator
VCO	Voltage-controlled oscillator
DSP	Digital signal processor
PIL	Processor-in-the-loop
EPWM	Enhanced pulse width modulator
THD	Total harmonic distortion
ADC	Analog-to-digital converter
SNR	Signal-to-noise ratio
ITAE	Integral of time-weighted absolute error
